# Supplementary material for: Shifting winter atmospheric teleconnections to the North Pacific reconcile Younger-Dryas and Holocene δ18O signals
Source: Nat Commun. 2026 Feb 6;17:2287. doi: 10.1038/s41467-026-68841-2 (PMC12976318; doi:10.1038/s41467-026-68841-2)
Supplement: Supplementary file 1 — Supplementary Information [file 41467_2026_68841_MOESM1_ESM.pdf]

Shifting Winter Atmospheric Teleconnections to the North Pacific reconcile Younger-Dryas and Holocene $\delta^{18}O$ signals

Lesleigh Anderson[1], Bruce P. Finney[2,3]*, W. Brad Baxter[3]
[1]United States Geological Survey, Geosciences and Environmental Change Science Center, Denver, CO, USA

[2]Idaho State University, Department of Biological Sciences, Pocatello, ID, USA.

[3]Idaho State University, Department of Geosciences, Pocatello, ID, USA.

*corresponding author: finney@isu.edu

Supplementary Methods and Discussion

Disclaimer: Any use of trade, firm, or product names is for descriptive purposes only and does not imply endorsement by the U.S. Government.

Study lake water characteristics and bathymetry

Surface water and water-column measurements for April Fools Lake include temperature, pH, specific conductance, and dissolved oxygen, and were collected with a calibrated Hydrolab Quanta™ sonde on April 1, 2015, and October 2, 2019. Note that summer data is not available for April Fools Lake. McMillan (2016)[1] provide temperature, pH, dissolved oxygen, and specific conductance for Neklason Lake (n=477) and Finger Lake (n=981) measured between 1998-2000. Note that several sediment cores are from shallow to moderate water depths and thus do not experience the full range of lake temperature, pH, specific conductance and DO conditions.

Lake Bathymetry was determined from GPS-located sonar-measured water depths at April Fools Lake (n=36) and Neklason Lake (n= 55). Finger Lake bathymetry was determined by digitizing depth data provided by Alaska Department of Fish and Game (www.adfg.alaska.gov). The data were mapped on a 2-meter grid cell bare earth digital terrain model derived from airborne light detection and ranging (lidar) data acquired by the Alaska Division of Geological and Geophysical Surveys (https://elevation.alaska.gov) and contoured using a kriging method of interpolation within GIS.

Supplementary Table 1: Lake parameters of April Fools Lake, Neklason Lake, and Finger Lake

Parameter	April Fools[2]	Neklason[1]	Finger[1]
Hydrologic configuration	Open<br>Groundwater fed with one surface outlet	Open<br>Groundwater fed with one surface inlet and outlet	Closed<br>Groundwater fed with no surface inlet or outlet
Thermal stratification	Seasonal	Seasonal	Seasonal
Field measurements			
Temperature range (˚C)	0.2-8	3.4-22.2	1.7-21.5
pH	8.6-9.2	8.0-8.8	6.5-9.8
Specific conductance (µS/cm)	162-305	181-328	129-677
Dissolved oxygen (mg/L)	0.8-1.44	<0.1-22.13	<0.1-15.9
Surface area (ha)	16.3	29.1	146.5
Max depth (m)	3.5	17.4	13.4

[1]Annual ranges from McMillan (2016)[1].
[2]Values are for April 1, 2015, and October 1, 2019. An average summer temperature estimate is ~16˚C.

Oxygen and hydrogen isotopes of water

Lake water samples for isotope analyses from April Fools Lake, Neklason Lake, and Finger Lake were obtained for this study in 30 ml HDLP Nalgene™ bottles with no headspace. Water samples were filtered and subsequently analyzed at Idaho State University on a Picarro™ I2130-I analyzer equipped with a vaporizer using an autosampler. All samples were measured six times. The first three injections were rejected to avoid the influence of sample memory and the last three injections were averaged to generate the reported value[2,3]. We analyzed three United States Geological Survey standards (USGS 46, USGS 47, USGS 48) that extended beyond the range of sample isotopic compositions at the beginning and end of each run for calibration, with standards run throughout the run to assess drift. Isotope values of $\delta^{18}O$ and $\delta^{2}H$ are reported as per mil (‰) relative to international standard VSMOW (Vienna Standard Mean Ocean Water) defined by $\delta^{18}O_{H2O} = [(^{18}O/^{16}O)_{H2O} / (^{18}O/^{16}O)_{VSMOW}] - 1$ and $\delta^{2}H_{H2O} = [(^{2}H/^{1}H)_{H2O} / (^{2}H/^{1}H)_{VSMOW}] - 1$. Uncertainty assessed by repeated analysis of the standards is <0.2‰ and <1.0‰ for oxygen and hydrogen, respectively.

We compiled oxygen and hydrogen isotope ratios of water from Matanuska-Susitna Valley (MatSu) hydrologic systems to compare with our study lake waters (Supplementary Fig. 1). They include precipitation[4], springs[5], groundwater[5] and other lakes[1]. Precipitation data from Anchorage (2005-2018) form a local meteoric water line (LMWL) with a slightly lower slope (7.15) than the Global Meteoric Water Line (GMWL). Surface water values from lakes defines a local evaporation line (LEL) with a slope of ~4. Lakes hydrologically closed to surface outflow and with long water residence times are strongly influenced by evaporation as indicated by relatively high $\delta^{18}O$ values up to ~-6‰.

For open lakes such as April Fools Lake and Neklason Lake, lower $\delta^{2}H$ and $\delta^{18}O$ values ranging between -17 and -15‰ plot near the intersection of the LEL and LMWL and groundwater values, which reflects their insensitivity to evaporation. The intersection point of the LEL and LMWL defines the average isotope values for unmodified mean annual precipitation (~-16‰) verified here by precipitation and groundwater data[5]. These results taken together, suggest that groundwater fed April Fools Lake and stream fed Neklason Lake act similarly as evaporation-insensitive lakes despite their slight differences in surface inflow configurations (Supplementary Table 1). Sub-surface inflow at April Fools Lake must be rapid enough to prevent significant evaporative enrichment in heavy isotopes. Therefore, both lakes are good representatives of regional groundwater $\delta^{18}O$ values and thus $\delta^{18}O$ of precipitation.

Closed lakes with hydrologic balances that are sensitive to water loss by evaporation, such as Finger Lake, have $\delta^{18}O$ values between -12‰ and -6‰. Although Finger Lake is groundwater fed like April Fools and Neklason lakes, it has no surface outflow and its extended lake water residence times lead to the preferential evaporation of light ^{16}O-water that leaves remaining lake water ^{18}O-enriched by ~8‰. This evaporation effect is notably large compared with multi-annual changes in $\delta^{18}O$ of precipitation and groundwater.

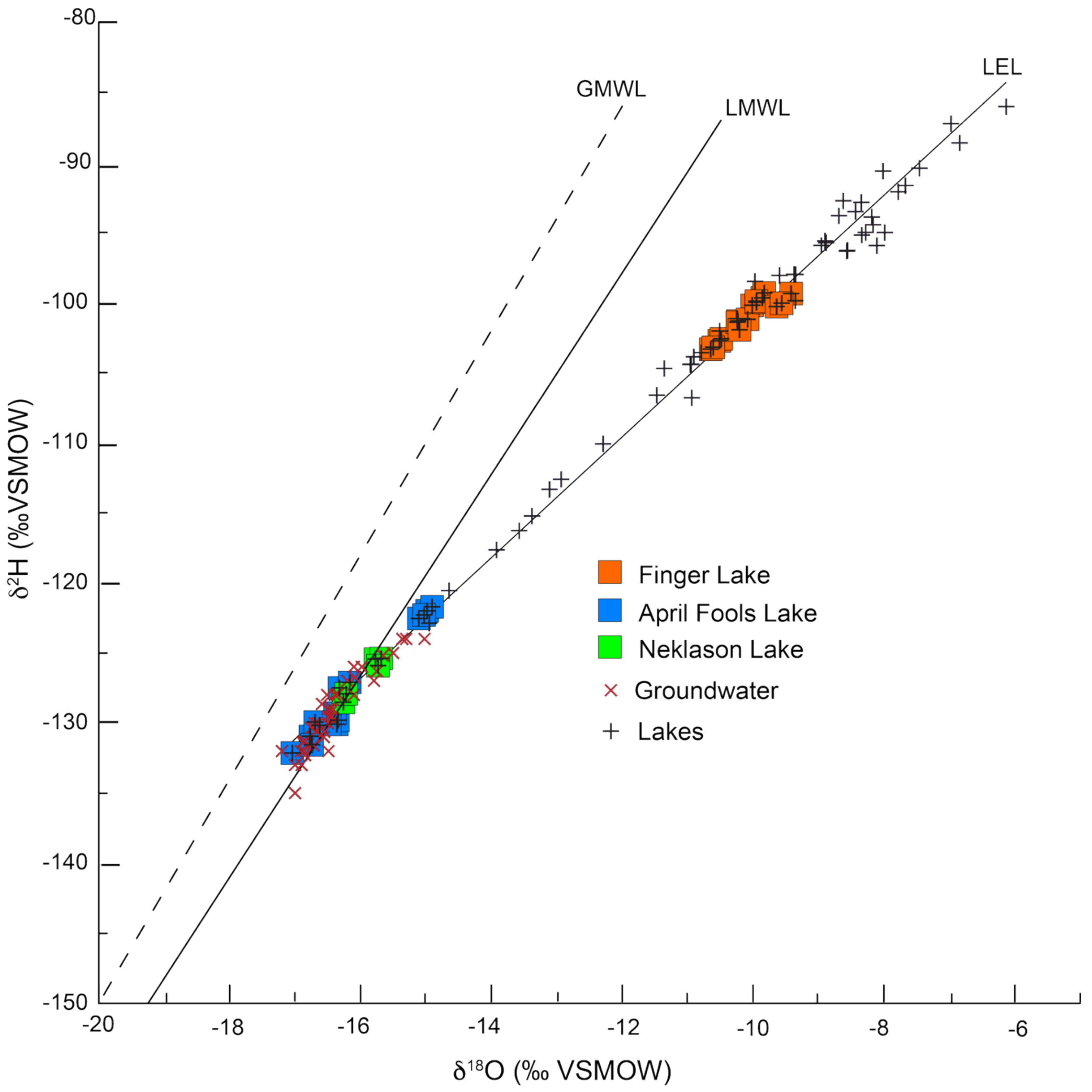

Supplementary Figure 1. Water isotope biplot of Matanuska-Susitna Valley hydrologic systems. April Fools Lake and spring inflow data are for 2015, 2016 and 2019 (blue squares; this study). Matanuska-Susitna Valley groundwater data is from 1999 and 2011 (red x symbol;)[5]. Finger Lake (orange squares), Neklason Lake (green squares), and other regional lakes (black + symbol)[1] form a lake evaporation line (LEL; slope of 4.3). Precipitation data (not shown) forms a Local Meteoric Water Line[4] (LMWL, slope of 7.15) to the right of the Global Meteoric Water Line (GMWL, slope of 8),

Sediment Cores

After whole core magnetic susceptibility measurements were conducted at 1-cm increments using a Bartington™ susceptibility meter, each core section was split, photographed, and visually logged for biogenic features, sedimentary structures, composition, and Munsell color. Contiguous volumetric samples were obtained at 1 cm increments for dry bulk density. Sample subsets at 2 to 6-cm increments were analyzed for percent organic matter and carbonate by Loss on Ignition (%LOI) measurements at 550˚C and 1000˚C[6,7].

Supplementary Table 2: Summary of lake and sediment core information

Lake/core name	Latitude (˚N)	Longitude (˚W)	Elevation (m)	Water depth (m)	Core length (cm)
April Fools A15	61.49191	149.99840	41	1.65	540
April Fools B15	61.48995	150.00032	41	3.25	775
Neklason A16	61.62828	149.27377	128	1.30	285
Neklason B16	61.62756	149.27393	128	1.22	385
Finger A16/23	61.60810	149.26459	103	1.60	705

Oxygen and carbon isotopes of sediment carbonate

Dried powdered carbonate samples were analyzed on a Finnigan GasBench II interfaced to a Delta V Advantage mass spectrometer through the ConFlo™ IV system at the Idaho State University Stable Isotope Laboratory, Pocatello, ID, United States. Samples were loaded into 12mm exetainer glass vials and reacted with phosphoric acid on a heating block at 72°C until the reaction was complete. Isotope values of $\delta^{13}C$ and $\delta^{18}O$ are reported as per mil (‰) values relative to the international standard Vienna Pee Dee Belemnite VPDB scale (VPDB) defined by $\delta^{18}O_{CaCO3} = [(^{18}O/^{16}O)_{CaCO3} / (^{18}O/^{16}O)_{VPDB}] - 1$ and $\delta^{13}C_{CaCO3} = [(^{13}C/^{12}C)_{CaCO3} / (^{13}C/^{12}C)_{VPDB}] – 1$. Four in-house standards (Carrara, Fisher, Merck and Calcite 1), which are directly calibrated using international standards (NBS-18, NBS-19, and LSVEC), were used to create a two-point calibration curve to correct the raw data and to monitor the accuracy of the data. Precision for both $\delta^{13}C$ and $\delta^{18}O$ is less than ±0.2‰. Prior to analyses, bulk samples were wet sieved through nested screens (250, 125, 63, 32 µm), collected separately and freeze-dried. The finest fraction (<32 µm) was analyzed and insignificant $\delta^{18}O$ differences occur among smaller grain sizes (Supplementary Table 3). All samples were visually examined for purity and homogenized before stable isotope analysis.

To verify an authigenic origin of the sediment carbonates, we applied to two approaches to test for carbonate isotopic equilibrium of surface sediments using equations that 1) predict equilibrium carbonate isotope composition from lake-water isotopic composition and temperature[8] and 2) predict lake-water temperature from carbonate in isotopic equilibrium with lake-water[9,10] (Supplementary Table 4). Using the first approach for our open lakes (April Fools and Neklason), the difference between measured and equilibrium carbonate $\delta^{18}O$ values is <0.2‰, supporting an authigenic origin. For our closed lake (Finger), the difference is also supportive but larger (-1.52‰), possibly due to the near-shore coring location in a shallow bay with higher lake water temperatures than estimated from the open lake and proximal to points of spring discharge that could locally lower lake water $\delta^{18}O$ values. Using the second approach, an authigenic carbonate origin at all our lakes is supported by predicted lake-water

temperatures for isotopic equilibrium of authigenic carbonates that are within the measured range (Supplementary Table 1, Supplementary Fig. 2).

Supplementary Table 3: Comparison of $\delta^{18}O$ and $\delta^{13}C$ values of living *Chara* calcite encrustations and associated bulk carbonate fine-grained surface for sediment size fractions in April Fools Lake.

Sample location	Material – size fraction (µm)	$\delta^{18}O$ (‰VPDB)	$\delta^{13}C$ (‰VPDB)	Maximum Δ–$\delta^{18}O$
Lake at A15 coring location	Living Chara-125	-15.60	-9.31	
	Living *Chara*-63	-15.64	-9.15	
	Living *Chara*-32	-15.78	-9.47	0.17
Lake at A15 coring location	Bulk carbonate-250	-15.69	-5.53	
	Bulk carbonate-125	-15.80	-5.50	
	Bulk carbonate-63	-15.63	-5.54	
	Bulk carbonate-32	-15.59	-5.85	0.21
Lake at northern shoreline	Living *Chara*-250	-16.59	-9.33	
	Living *Chara*-125	-16.30	-7.80	
	Living *Chara*-63	-16.50	-8.38	
	Living *Chara*-32	-16.84	-9.04	0.54
Lake at northern shoreline	Bulk carbonate-250	-15.98	-5.60	
	Bulk carbonate-125	-16.13	-6.08	
	Bulk carbonate-63	-16.06	-6.22	
	Bulk carbonate-32	-16.41	-6.99	0.42
Artesian spring NE shoreline	Living *Chara*-250	-17.66	-8.94	
	Living *Chara*-125	-17.00	-10.12	
	Living *Chara*-63	-16.95	-9.24	0.71
Artesian spring NE shoreline	Bulk carbonate-250	-16.35	-11.28	
	Bulk carbonate-125	-16.28	-10.80	
	Bulk carbonate-63	-16.53	-11.28	
	Bulk carbonate-32	-16.58	-10.91	0.30

Supplementary Table 4: Comparison of measured and predicted $\delta^{18}O$ values of lake sediment core surface sample bulk fine-grained (<32µm) carbonate

Site Name	Measured $\delta^{18}O_{Calcite}$ (‰VPDB)	Measured $\delta^{18}O_{Water}$ (‰VSMOW)	Estimated[1] maximum lake water temperature (˚C)	Predicted[2] equilibrium $\delta^{18}O_{Calcite}$ (‰VPDB)	$\Delta\ \delta^{18}O_{Calcite}$ (measured – predicted) (‰VPDB)	Predicted[3] equilibrium lake water temperature (˚C)
April Fools Lake	-15.54	-15.38	16	-15.38	-0.16	13.8
Neklason Lake	-16.47	-16.32	18	-16.32	-0.15	14.3
Finger Lake	-13.54	-12.02	21	-12.02	-1.52	17.9

[1]McMillan (2016)
[2]Friedman and O'Neil (1977)[8]
[3]Kim and O'Neil (1997)[9] and Leng and Marshall (2004)[10]

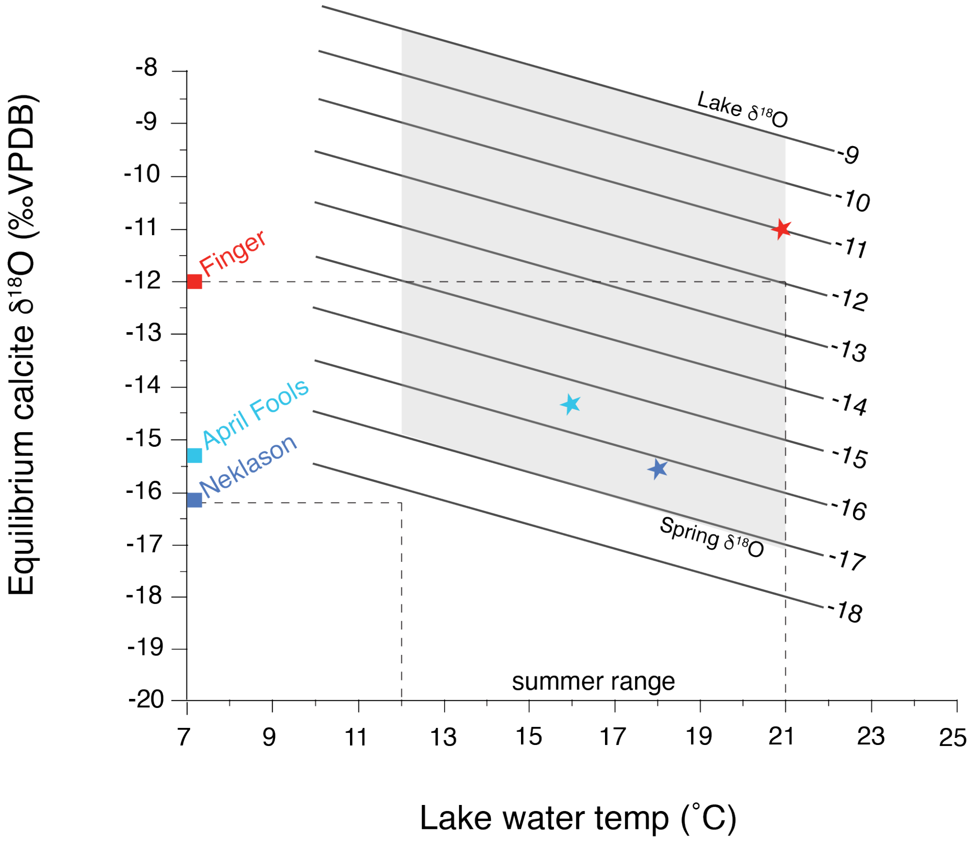

Supplementary Figure 2: Equilibrium calcite $\delta^{18}O$ are shown versus lake water temperature as a function of lake water $\delta^{18}O$[8]. On the horizontal axis, the summer range of lake water temperatures is delineated by dashed vertical lines. On the vertical axis, colored squares are measured carbonate $\delta^{18}O$ and horizontal dashed lines delineate those measured values. The gray area reflects the range of lake-water $\delta^{18}O$ within the summer lake water temperature range (Supplementary Table 1). Colored stars are the average measured lake-water $\delta^{18}O$ plotted by estimated average lake-water summer temperature.

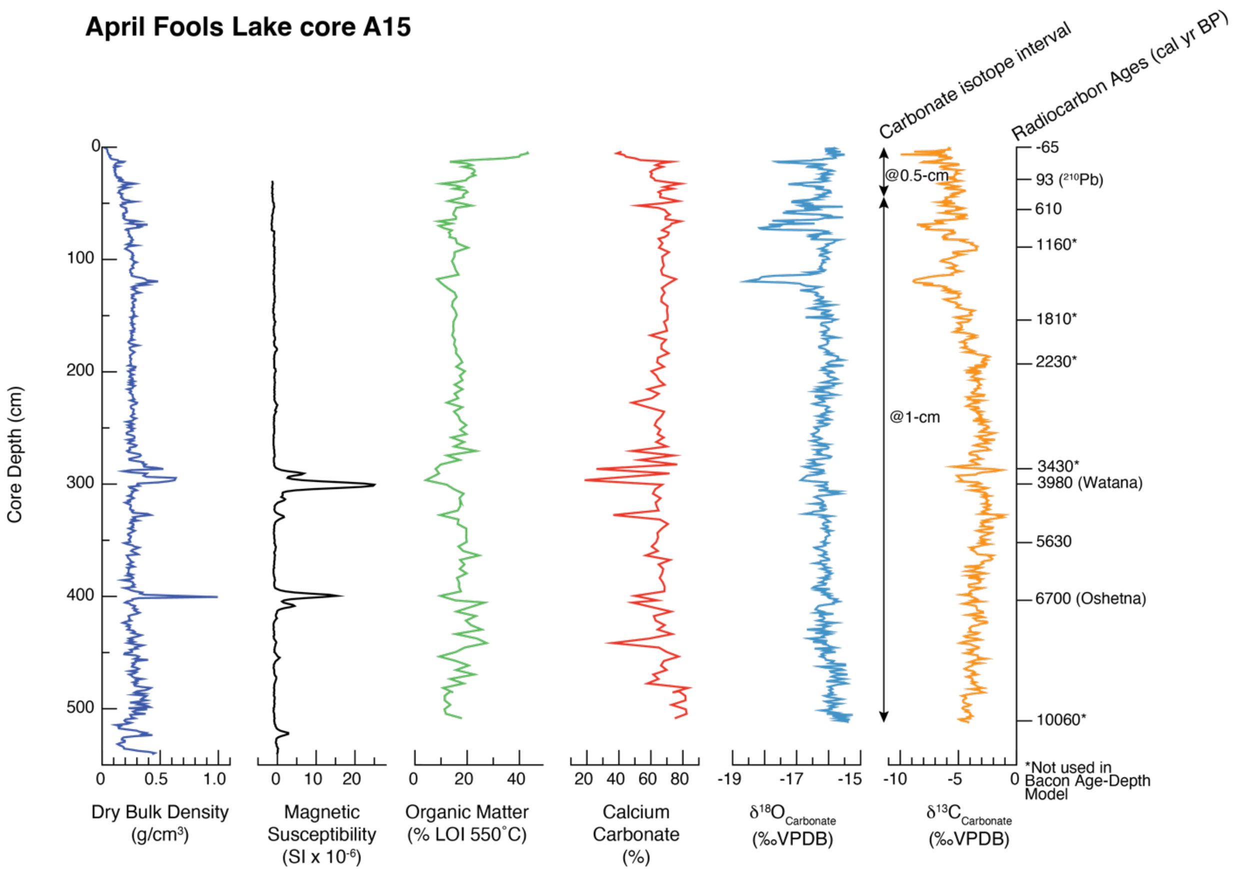

Supplementary Figure 3a: April Fools Lake core A15 sediment data on a vertical depth scale: dry bulk density, magnetic susceptibility, organic matter and percent calcium carbonate (based on LOI), carbonate oxygen and carbon isotopes, and calibrated radiocarbon ages. Vertical arrows indicate carbonate isotope sample intervals.

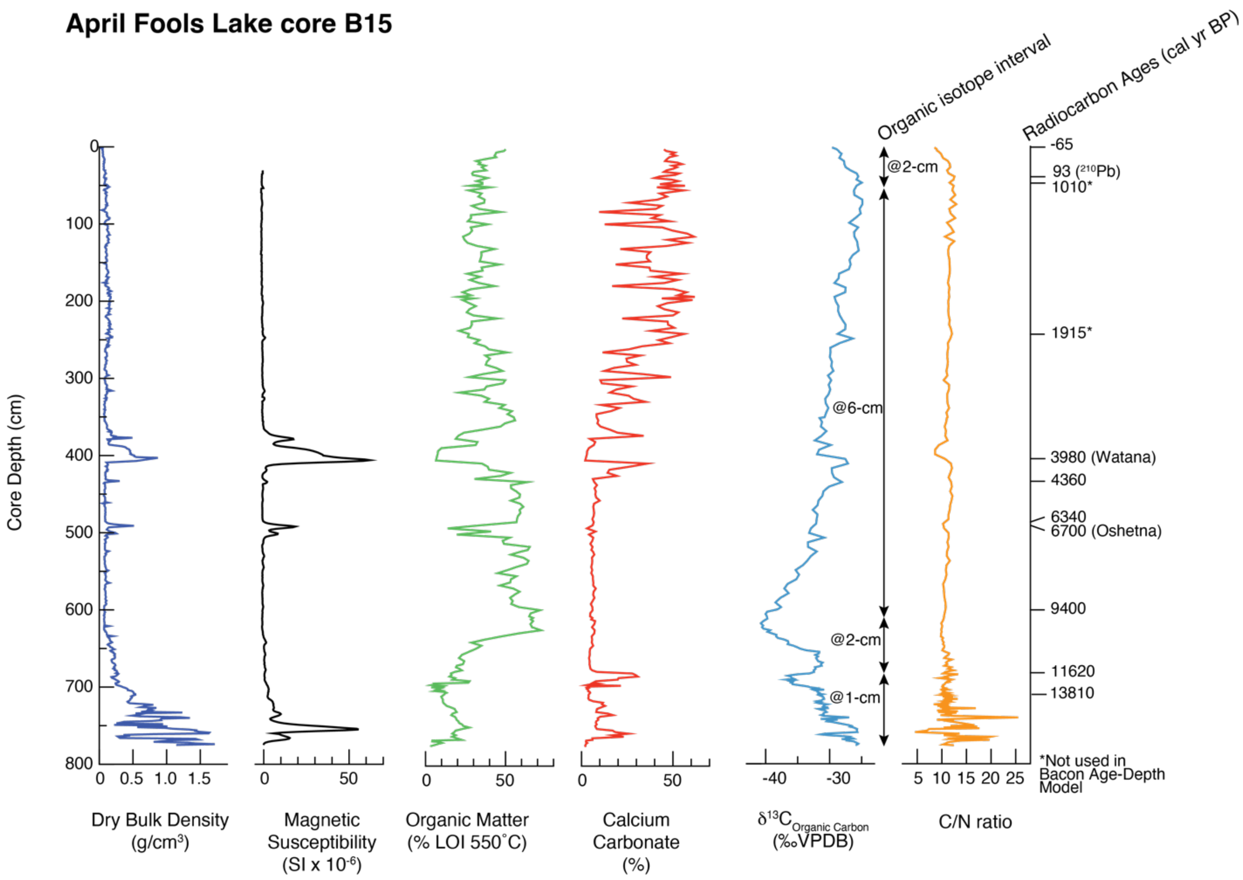

Supplementary Figure 3b: April Fools Lake core B15 sediment data on a vertical depth scale: dry bulk density, magnetic susceptibility, organic matter and percent calcium carbonate (based on LOI), organic carbon isotopes and C/N ratios, and calibrated radiocarbon ages. Vertical arrows indicate organic carbon isotope sample intervals.

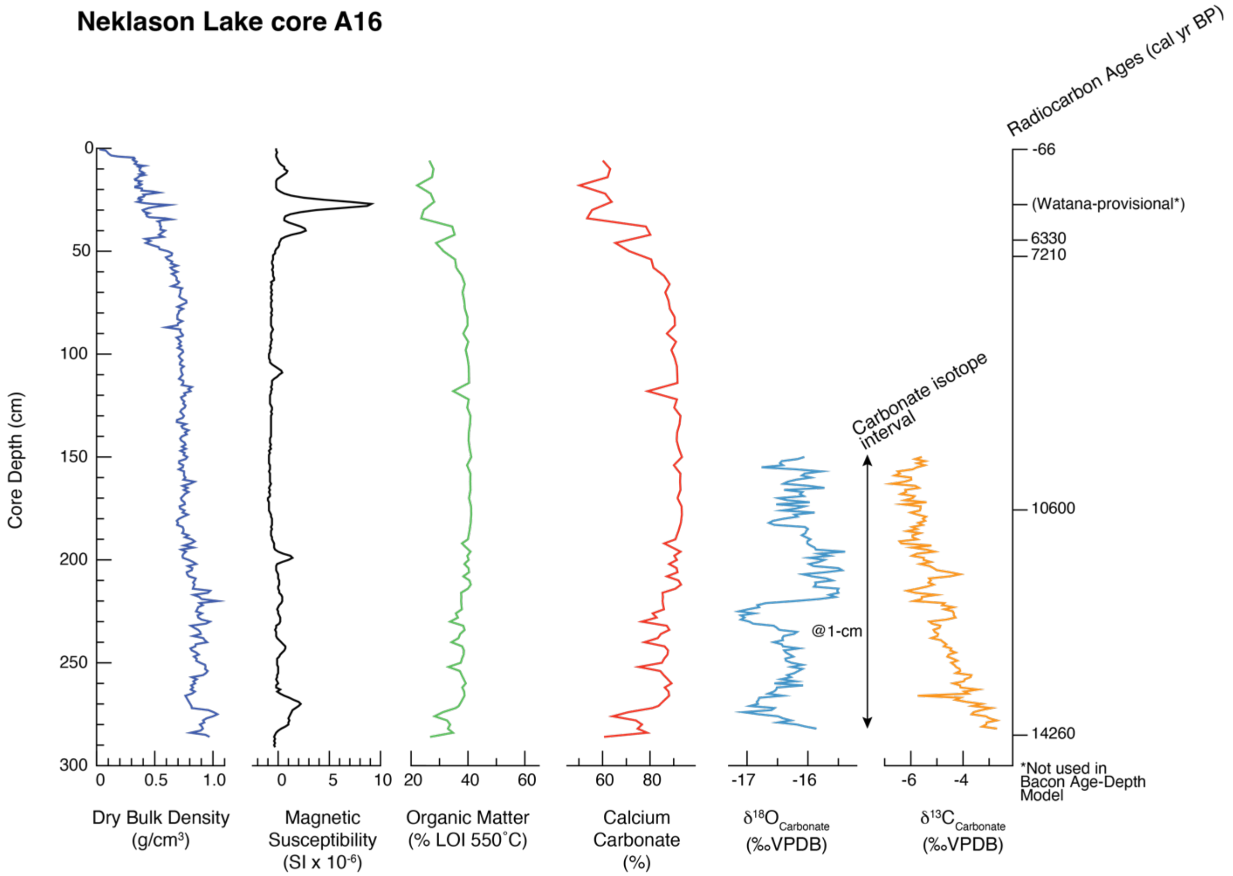

Supplementary Figure 3c: Neklason Lake core A16 sediment data on a vertical depth scale: dry bulk density, magnetic susceptibility, organic matter and percent calcium carbonate (based LOI), carbonate oxygen and carbon isotopes, and calibrated radiocarbon ages. Vertical arrow indicates carbonate isotope sample intervals.

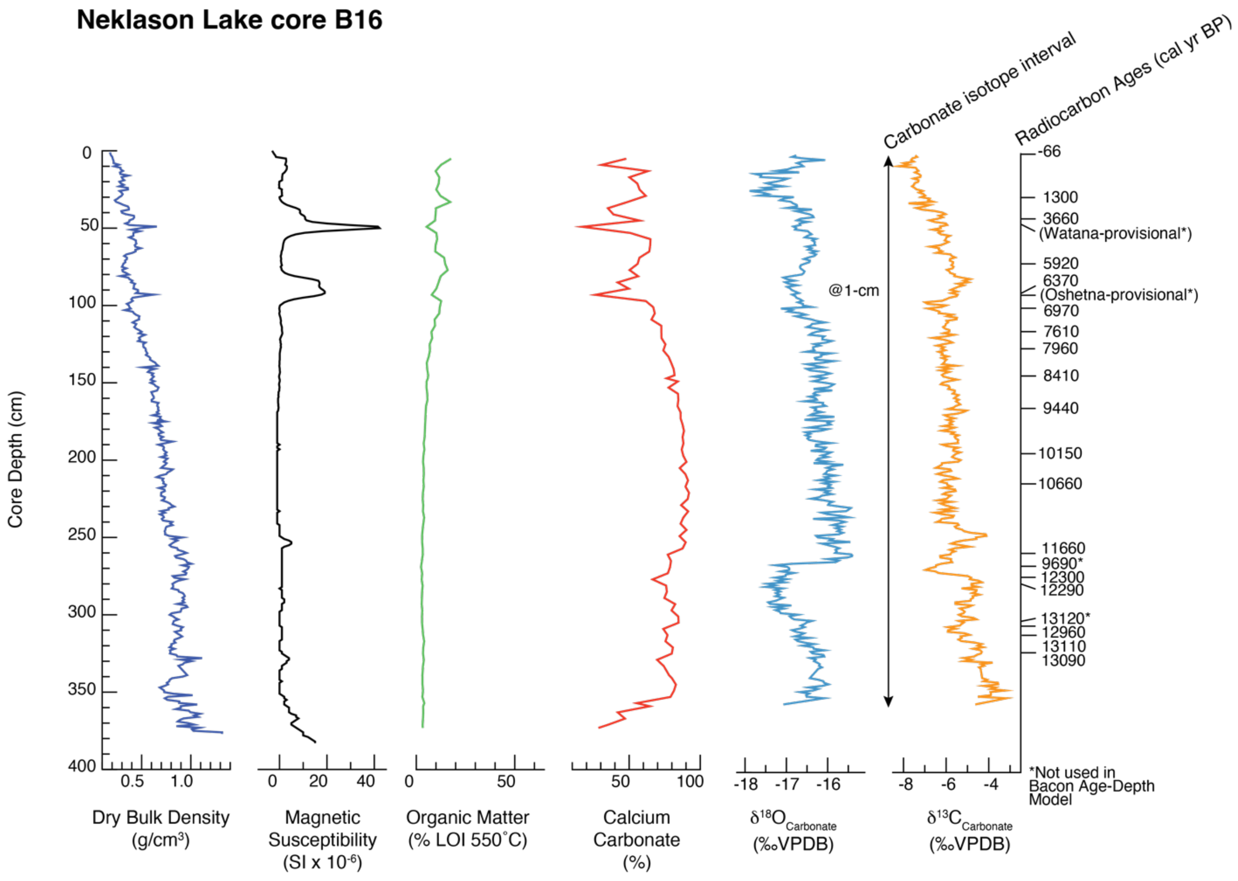

Supplementary Figure 3d: Neklason Lake core B16 sediment data on a vertical depth scale: dry bulk density, magnetic susceptibility, organic matter and percent calcium carbonate (based on LOI), carbonate oxygen and carbon isotopes, and calibrated radiocarbon ages. Vertical arrow indicates carbonate isotope sample intervals.

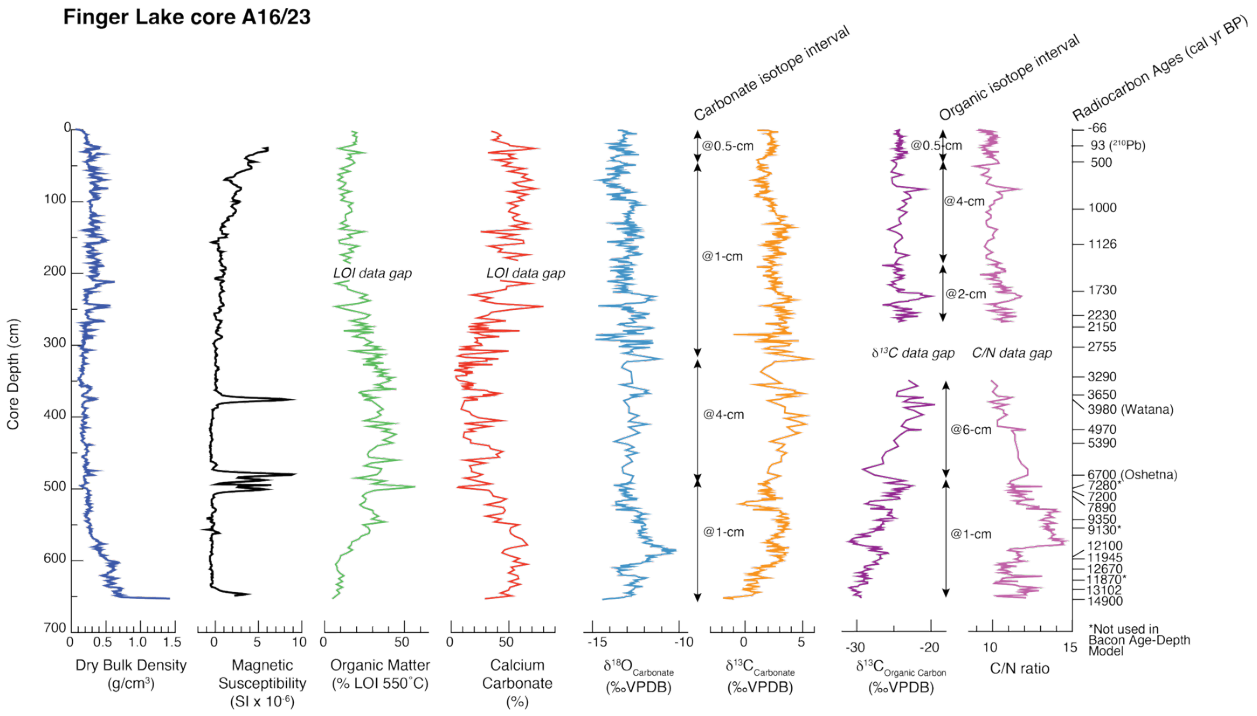

Supplementary Figure 3e: Finger Lake core A16/23 sediment data on a vertical depth scale: dry bulk density, magnetic susceptibility, organic matter and percent calcium carbonate (based on LOI), carbonate oxygen and carbon isotopes, organic carbon isotopes and C/N ratios, and calibrated radiocarbon ages. Vertical arrows indicate isotope sample intervals.

Sediment core chronologies

Lead-210

Lake surface sediments were measured for radiogenic ^{210}Pb on 0.5-cm thick sample intervals at the St. Croix Research Station of the Science Museum of Minnesota. These samples were derived from cores contiguously extruded at 0.5-cm increments from the surface to depths of 30- to 40-cm. For each chronology, a well-defined break referred to as the ^{210}Pb horizon occurs between sediment containing unsupported ^{210}Pb values lower than down-core supported ^{210}Pb (Supplementary Table 5). The ^{210}Pb-based ages were determined by the Constant Rate of Supply (CRS) age-depth model[11]. Well-defined declines in unsupported ^{210}Pb extend to 40-cm depth at April Fools Lake and to 21-cm depth at Finger Lake indicate clear ^{210}Pb horizons and provide robust chronologies for the past ~150 years. Total ^{210}Pb amounts in Neklason Lake are relatively low and unsupported ^{210}Pb extends to less than 10-cm depth, which reflect the low sedimentation rates in the shallow water environment.

Supplementary Table 5: Lead-210 data and ages

Lake - Core	Core Depth Mid of interval (cm)	Total ^{210}Pb (pCi/g)	^{210}Pb Year AD	^{210}Pb Year AD Error (± SD)
April Fools A15	0.25	3.435	2015.2	0.70
	3.25	3.414	2013.2	0.72
	6.25	3.413	2009.3	0.77
	9.25	3.524	2004.5	0.84
	11.75	3.927	1999.4	0.90
	14.25	3.117	1992.8	0.77
	15.75	2.876	1987.9	0.83
	17.75	2.792	1980.4	0.97
	19.25	2.170	1974.5	0.95
	20.75	1.851	1969.0	1.02
	22.75	1.891	1961.1	1.24
	24.25	1.622	1954.5	1.41
	26.25	1.280	1945.0	1.49
	28.25	1.091	1934.6	1.81
	29.25	0.986	1929.1	2.12
	30.75	0.700	1920.0	2.39
	32.75	0.518	1906.1	2.67
	34.25	0.362	1894.8	3.01
	35.75	0.333	1883.6	4.17
	37.25	0.270	1872.6	5.63
	38.75	0.246	1860.9	8.10
	40.25	0.185	1847.4	11.88

Supported ^{210}Pb = 0.1058 ± 0.0006 pCi/g, number of supported samples = 2

Lake - Core	Core Depth Mid of interval (cm)	Total ^{210}Pb (pCi/g)	^{210}Pb Year AD	^{210}Pb Year AD Error (± SD)
April Fools B15	0.25	2.803	2015.2	1.78
	5.25	3.804	2010.1	1.79
	7.75	3.712	2004.1	1.99
	10.25	3.092	1995.9	2.25
	12.25	2.011	1986.1	2.08
	14.25	1.425	1975.4	1.99
	16.25	1.149	1965.6	2.22
	18.25	0.924	1955.7	2.57
	20.25	0.732	1945.7	3.10
	22.25	0.513	1935.3	3.65
	24.25	0.427	1924.4	4.86
	26.25	0.318	1911.8	6.72
	28.25	0.306	1891.0	13.57
	30.25	0.147	1860.2	26.16

Supported ^{210}Pb = 0.0718 ± 0.0091 pCi/g, number of supported samples = 7

Lake - Core	Core Depth Mid of interval (cm)	Total ^{210}Pb (pCi/g)	^{210}Pb Year AD	^{210}Pb Year AD Error (± SD)
Neklason C19	0.25	1.150	2018.7	1.34
	1.25	1.193	2013.5	1.48
	2.25	1.176	2006.2	1.72
	3.25	1.031	1996.5	2.15
	3.75	0.677	1991.0	2.46
	4.75	0.527	1980.2	3.09
	5.75	0.377	1969.3	3.75
	6.25	0.380	1963.6	4.48
	7.25	0.410	1945.3	8.55
	8.25	0.250	1918.2	14.21
	9.25	0.192	1894.7	24.74

Supported ^{210}Pb = 0.1676 ± 0.0075 pCi/g, number of supported samples = 5

Lake - Core	Core Depth Mid of interval (cm)	Total ^{210}Pb (pCi/g)	^{210}Pb Year AD	^{210}Pb Year AD Error (± SD)
Finger A16	0.25	0.930	2015.9	0.0034
	3.25	0.500	2009.2	0.0037
	6.25	0.424	2001.0	0.0037
	8.25	0.446	1993.6	0.0026
	9.75	0.368	1987.2	0.0033
	11.25	0.373	1980.3	0.0028
	13.25	0.332	1969.0	0.0028
	14.75	0.313	1958.1	0.0026
	15.75	0.266	1949.5	0.0033
	16.75	0.256	1939.8	0.0033
	17.75	0.207	1928.8	0.0047
	18.75	0.204	1916.2	0.0046
	19.75	0.172	1901.1	0.0067
	20.25	0.177	1892.0	0.0061
	21.75	0.132	1857.0	0.0179

Supported ^{210}Pb = 0.1106 ± 0.0076 pCi/g, number of supported samples = 4

Tephra

We identified two volcanic tephra layers as originating from Mt. Hayes, Alaska based on a range of evidence. Two visible tephra layers in April Fools Lake cores are a similar light tan/buff gray color, dense in texture, with thicknesses between 1-3 cm. Two similarly sharp magnetic signatures among April Fools Lake, Neklason Lake and Finger Lake correlate on depth scales (Supplementary Fig. 4). The chronostratigraphic positions of the two tephras based on our radiocarbon data confirms their identification as the only two prominent regional tephra layers that have been determined to be marker horizons in the region, originating from the Mt. Hayes volcano[12]. They are identified as the Watana tephra with an age of 3650 ± 150 ^{14}C yr BP (3980 cal yr BP, one sigma range of 3360 to 4400 cal yr BP) and the informally named Oshetna tephra documented in the Susitna Valley at 5875 ± 85 ^{14}C yr BP (~6700 cal yr BP, one sigma range of 6570 to 7930 cal yr BP). These combined lines of evidence allow us to have confidence in our tephra identification and application of their independently pre-determined age to our radiocarbon age-depth models for April Fools Lake and Finger Lake cores described below.

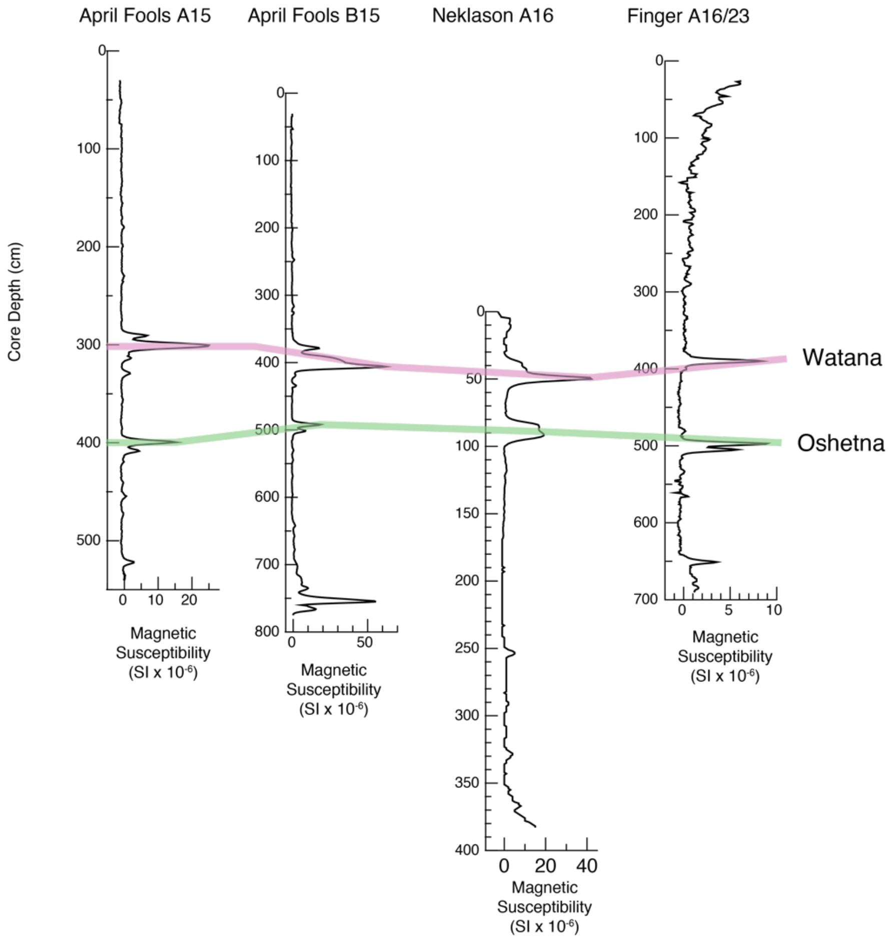

Supplementary Figure 4: Magnetic susceptibility peak correlations identified as Watana and Oshetna tephras from Mt. Hayes, Alaska in April Fools Lake, Neklason Lake, and Finger Lake sediment cores.

Radiocarbon

Radiocarbon chronologies are based on accelerator mass spectrometry (AMS) ^{14}C measurements of graphitized macrofossils made at the Woods Hole Oceanographic Institution (WHOI) National Ocean Sciences Accelerator Mass Spectrometry (NOSAMS) laboratory in Woods Hole, MA, (NOSAMS lab numbers), the Lawrence Livermore National Laboratory, Livermore, CA (CAMS lab numbers), and the U.S. Geological Survey Radiocarbon Laboratory, Lakewood, CO (USGS lab numbers). For selected depths in April Fools Lake where no terrestrial material was available, aquatic material was dated and is discussed below. Both measured and calibrated radiocarbon ages (Calib 8.2)[13] are reported but only calibrated ages (cal yr BP; 1950, also referred to here as 'ka') are used for discussion (Supplementary Table 7). Mean age-depth models were generated using core-top ages, ^{210}Pb chronologies, independent tephra ages, and terrestrial macrofossil ^{14}C ages using the Bayesian software Bacon[14] with the most recent calibration curve (IntCal 20) using default prior assumptions.

April Fools Lake

The April Fools Lake core A15 age-depth model is based on the surface age, ^{210}Pb, three ^{14}C dates of terrestrial macrofossils, and two Mt. Hayes tephra layers, Watana and Oshetna (Supplementary Fig. 5a, upper panel). The oldest reliable age is the Oshetna tephra and the extrapolated age to the base of marl sediments is ~10 ka. Estimated sedimentation rates are 0.4 to 1.5 mm/yr, for an average of 17 yrs/cm. The average age uncertainty of the record is 372 ± 218 yrs (min = 30 yrs, max = 915 yrs).

Terrestrial macrofossils were not present at most depths in April Fools Lake core A15 and aquatic macrofossils were measured. The resulting aquatic ages were in stratigraphic order but consistently older than the depth relationship established by the tephra and terrestrial macrofossil ages. An aquatic macrofossil found within the Oshetna tephra layer produced a ^{14}C age 3400 years older than the independent tephra age that we used to estimate the lake's old-carbon reservoir effect. Although the application of this value as a reservoir correction aligns the remaining aquatic ages in a consistent stratigraphic manner with the tephra and terrestrial dates (Supplementary Fig. 3a), the uncertainty of the reservoir correction error through time is unknown. Because incorporating reservoir corrected aquatic ages maintains but does not improve the level of uncertainty of the terrestrial based age-depth models, we limited their use to validate and cross-check the Bacon age-depth models (Supplementary Fig. 5a, upper panel). This cautious approach suggests our age model is consistent with all available data.

The April Fools core B15 age model is based on the surface age, ^{210}Pb, and four ^{14}C dates of terrestrial macrofossils, and the two Mt. Hayes tephra layers, Watana and Oshetna (Supplementary Fig. 5a, lower panel). Estimated sedimentation rates are 0.2 to 2 mm/yr for an average of 23 yrs/cm. Rates are steady from ~14 and 8 ka and increase between 5ka to the core top. The oldest reliable date is 13.8ka, determined from wood. An additional sample from the same depth contained coal and returned an age of 40ka and was excluded. The extrapolated basal age is ~15ka. The average age uncertainty of the record is 473 ± 169 yrs (min = 4 yrs, max = 765 yrs).

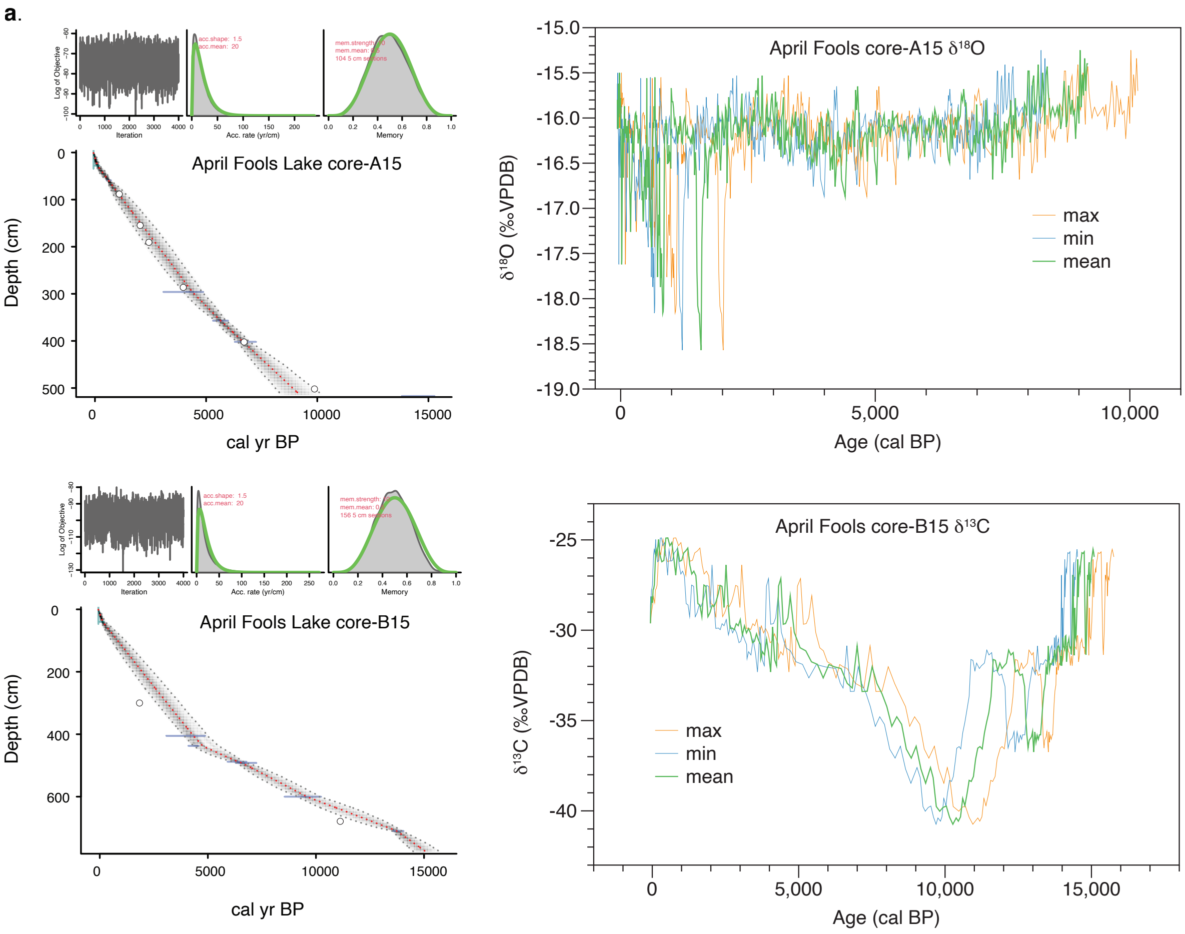

Supplementary Figure 5a. April Fools Lake Bayesian age-depth models shown with proxy plots for minimum (blue), maximum (orange), and mean (green) ages within 95% confidence limits (core A15 carbonate δ^{18}O, upper panel, and core B15 organic δ^{13}C, lower panel). Reservoir-corrected aquatic ages for A15 (open circles, upper panel) are consistent with the terrestrial and tephra ages although not used in the Bacon model (see discussion).

Neklason Lake

The Neklason Lake core B16 age-depth model is based on the surface age, the ^{210}Pb horizon, and 18 ^{14}C dates on terrestrial macrofossils (Supplementary Fig. 5b, upper panel). Although both tephra layers occur in the upper meter of the sediment record (Supplementary Fig. 4), their inclusion does not improve the level of uncertainty of the terrestrial based age-depth models. Two radiocarbon ages were excluded that fell out of the age-depth trend defined by the other samples. One of these was a small sample (264 cm), and the other a wood fragment (304 cm) that was slightly out of sequence (Supplementary Table 7). The oldest dated depth is 13ka and the extrapolated basal age is ~14ka. Estimated sedimentation rates range from 0.1 to 0.5 mm/yr for an average of 38 yrs/cm. From ~13 to 6ka, sedimentation rates were high and steady. After 6ka, they abruptly declined and remained low up to present. The average age uncertainty of the record is 278 ± 246 yrs (min = 50 yrs, max = 791yrs). Correlation between abrupt shifts in Neklason Lake carbonate δ^{18}O during the YD chron to the Greenland ice core defined YD boundaries is strongest using the average age model within the 95% confidence limits.

Neklason A16 core carbonate isotope analyses were limited to the lower section for the purposes of verifying deglacial shifts in $\delta^{18}O$ during the deglacial as observed in core B15 (SI Fig. 5b, lower panel). The age-depth model is based on the surface age, the ^{210}Pb horizon, and four ^{14}C dates of terrestrial macrofossils and correlation of the $\delta^{18}O$ shifts to those in core B16 is strong using the average age model within the 95% confidence limits.

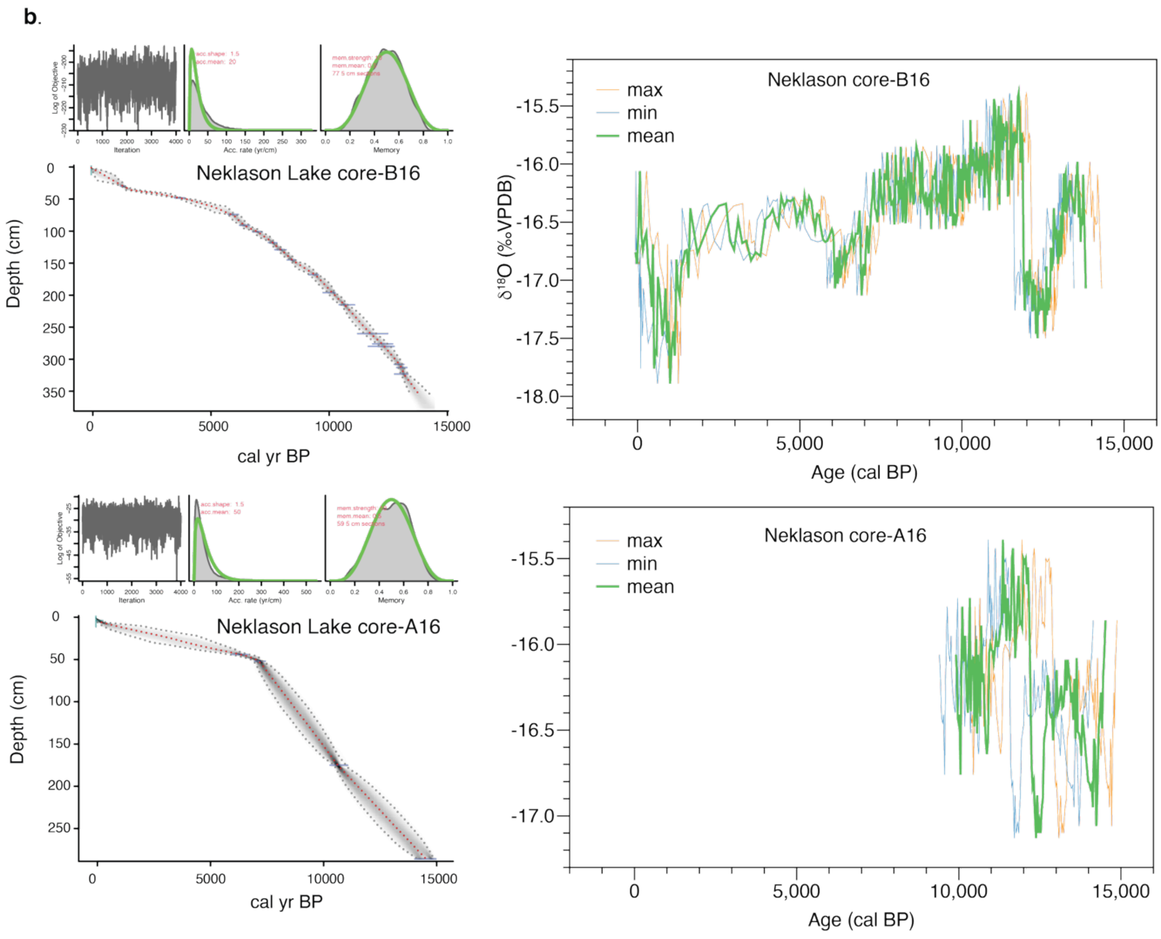

Supplementary Figure 5b. Neklason Lake Bayesian age-depth models shown with carbonate $\delta^{18}O$ plots for minimum (blue), maximum (orange), and mean (green) ages within 95% confidence limits (core B16, upper panel and core A16, lower panel). Core A16 $\delta^{18}O$ data shows replication of the YD signal by an independent core.

Finger Lake

The age model for Finger Lake composite core A16/23 is based on the surface age, ^{210}Pb, 21 ^{14}C dates of terrestrial macrofossils, and the two Mt. Hayes tephra layers, Watana and Oshetna (Supplementary Figure 5c). Three ages (492 cm 542 cm, 627 cm) that fell slightly out of the age-depth trend defined by the other samples were excluded due to small sample size (Supplementary Table 7). Estimated sedimentation rates are 0.25 to 2 mm/yr, for an average of 21 yrs/cm. Rates are high from ~14.5 to 12ka, slower between ~12 and 8ka, and increase from 6ka to present. The oldest date of 14.5ka is based on insect parts very near the base of the core and the calibrated range overlaps that of an extrapolated age from 13.2ka determined on a wood sample. The average age model uncertainty is 208 ± 139 yrs (min = 6yrs, max = 633yrs).

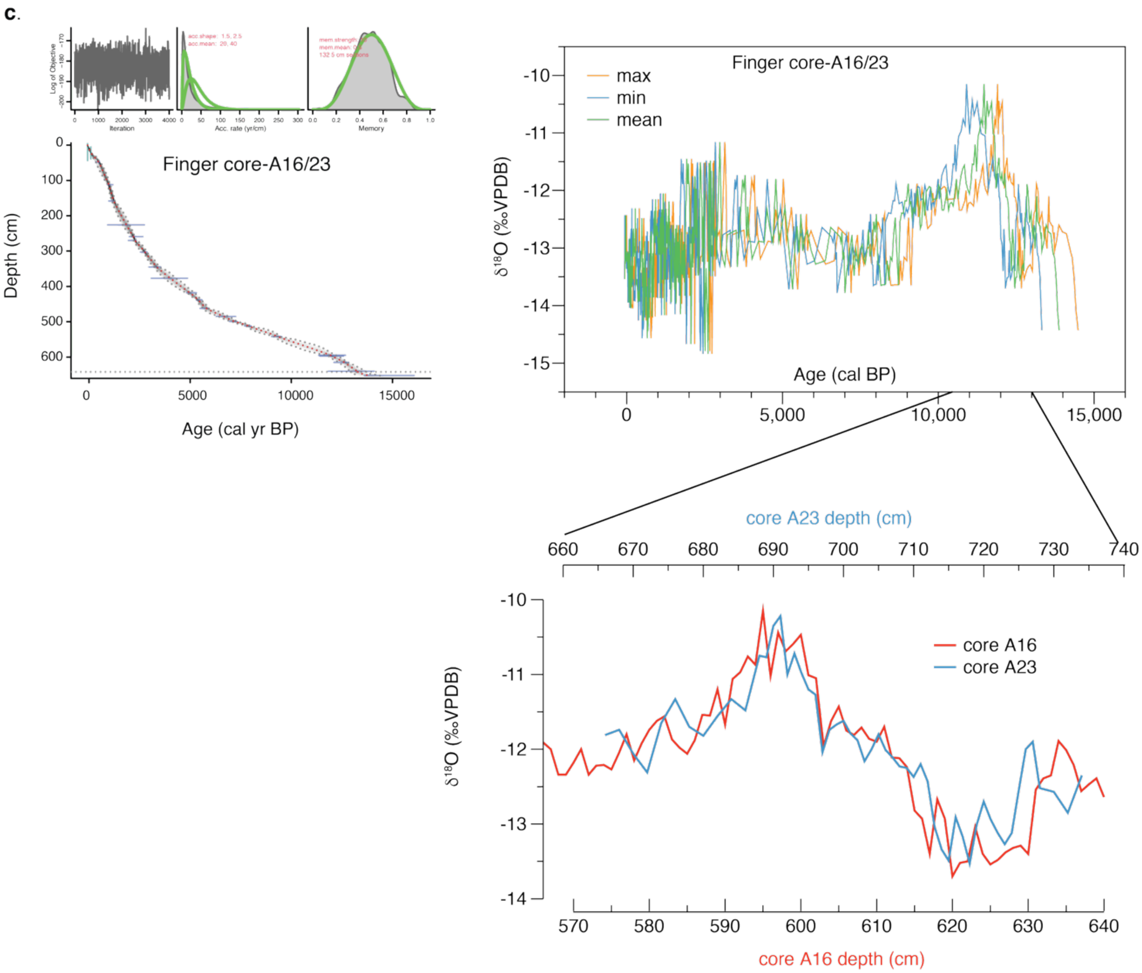

Supplementary Figure 5c. Finger Lake core A16/23 (upper panel) Bayesian age-depth model and carbonate δ^{18}O plots for minimum (blue), maximum (orange), and mean (green) ages within 95% confidence limits. The expanded depth scale plot across the YD chron (lower panel) shows that the δ^{18}O signal is well replicated by independent cores A16 and core A23.

Alaskan lake chronologies

The chronologies for the five other lake records used in the organic carbon isotope analyses (Fig 3) were developed from published age data. Age-depth models from Arolik Lake[15] and Dune Lake[16] were obtained from the original publications. We developed Bacon age-depth models for Birch Lake, Little Harding Lake, and Eightmile Lake, that are described below. A summary of the sediment records utilized in this study in shown in Supplementary Table 6, including length, dated depths, oldest dated depth, inferred or measured basal age, age-depth model uncertainty, sampling interval and average sedimentation rates.

The Birch Lake age model is based on 20 ^{14}C dates of terrestrial macrofossils and pollen for 15 depths (5 depths are paired pollen-macro ^{14}C samples) and core tops from two stratigraphically correlated cores from similar depocenter water depths (97B and 97G/H[17]). Estimated sedimentation rates range from 0.05 to 0.4 mm/yr, for an average 57 yrs/cm. The oldest date is 15ka and the extrapolated basal age is ~16.8ka. The estimated age uncertainty is 496 ± 174 yrs (min = 373 yrs, max = 479 yrs).

The Eightmile Lake age model is based on the published surface age and four ^{14}C dates from two terrestrial macrofossils and two pollen extracts from depocenter core PC2-07[18]. Estimated sedimentation rates range from 0.1 to 1.3 mm/yr, for an average of 89 yrs/cm. The oldest date is 13.9ka and is basal. The average age uncertainty is 912 ± 376 yrs (min = 111 yrs, max = 1314 yrs).

The Little Harding Lake age-depth model is based on the published surface age and five ^{14}C dates of terrestrial macrofossils and pollen from depocenter core Z[19]. Estimated sedimentation rates range from 0.2 to 0.6 mm/yr, for an average of 25 yrs/cm. The oldest date is ~14ka and is near basal. The average age uncertainty is 425 ± 117 yrs (min = 106 yrs, max = 609 yrs).

Supplementary Table 6: Summary of Mat-Su and Interior Alaska lake sediment core

Lake Core-Year	Core length (cm)	Water depth (cm)	Dated horizons (#)	Approx. oldest date (cal yr BP)	Est. basal age (cal yr BP)	95% Age model limits (cal yr BP)	Age model avg. error (cal yr BP)	Sample interval (cm)	Avg. sed resolution (yr/cm)
April Fools A15	540	164	6	6800	10,000	30-915	372 ± 218	0.5, 1	17
April Fools B15	775	325	7	13,800	15,000	4-765	473 ± 169	0.5, 1	23
Neklason B16	377	122	18	13,000	14,000	50-791	278 ± 246	0.5, 1	38
Finger A16/23	653	156	23	14,900	14,900	6-633	208 ± 139	0.5 - 6	21
Birch 97B	696	1290	15	15,000	16,800	373-479	496 ± 174	1	57
Eightmile PC2-07	163	280	6	13,900	13,900	111-1314	912 ± 376	1	89
L. Harding Z	579	830	11	14,000	14,000	106-690	425 ± 117	1	25
Dune A-F[1]	638	900	15	11,195	13,500	N/A	N/A	1	16
Arolik 2-6[2]	860	4000	39	35,000	35,000	N/A	N/A	5	37

[1]Lynch et al. (2002)[16]
[2]Kaufman et al. (2002)[15]

April Fools Lake Deglacial Pollen

Samples were analyzed for pollen from April Fools Lake core B15 for the interval between 9000 to 14,500 cal yr BP to investigate vegetation changes during the deglacial period. Pollen samples were processed using standard techniques[20]. In short summary, after adding a known quantity of exotic pollen (tablets of *Lycopodium clavatum* spores), samples underwent acid and base washes to remove carbonates, organics, and silica. Pollen preparations were mounted on slides with silicon oil and analyzed at 400x and 1,000x. Pollen identifications were made by comparison with the pollen reference collection housed at the University of Alaska Fairbanks, as well as comparisons with published pollen atlases. Pollen counts were conducted by Nancy Bigelow and diagrams were made with Tilia v.2.1.1[21].

During the Bølling-Allerød, herbs and forbs declined with rising *Betula*, aquatic spores, algae, and desmids, consistent with the shallow lake environment. The period after the Younger-Dryas and into the early Holocene is characterized by a *Betula* decline, with no change in herbs and forbs, and prominent rise in fern spores (summed monolete spores) and aquatic indicators, such as small desmids. Percentages of *Artemesia, Alnus, Salix* and *Populus* were too low (<10%) to identify changes. These subtle terrestrial vegetation trends are similar to other vegetation studies in the region that do not show a strong or consistent YD response[12]. The rise in *Picea* begins after ~10ka, following regional trends.

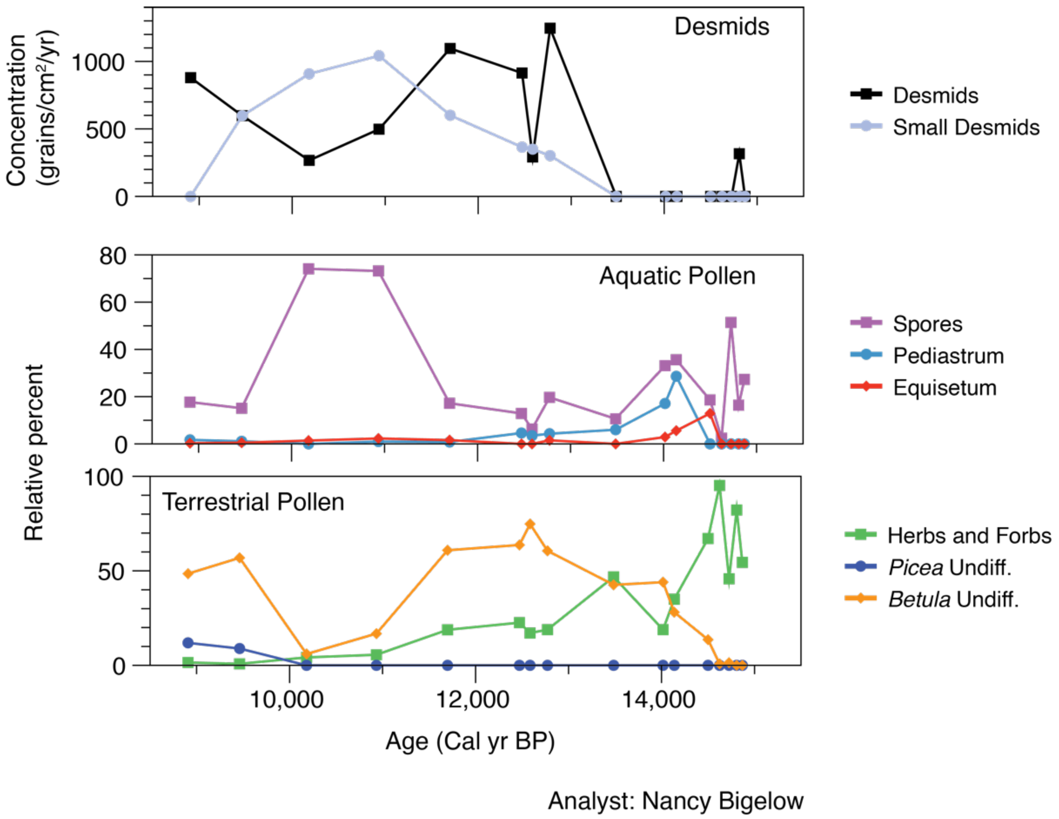

Supplementary Fig 6. April Fools Lake core B15 major pollen taxa (relative percent) between 9 and 14ka. Desmid types are shown as concentrations.

Composite April Fools Lake and Neklason Lake δ^{18}*O record*

The high resolution δ^{18}O record from April Fools core A15 extends to ~9ka and has chronological control to ~7ka. In contrast, the upper section of Neklason core B16, with chronological control throughout, is of low resolution during the past ~6ka and high resolution between ~6 and 14.5 ka (Supplemental Fig. 5b). To provide a single high-resolution stable isotope record, the data were merged based on stratigraphic δ^{18}O overlap between the cores (Supplementary Fig. 7). The overlapping portions of the δ^{18}O records show similar means and variance between -15.5 and -18.5‰. Given this, and the similarity in low-frequency trends of the late Holocene records, the independent chronologies were spliced at 7.6ka with no scaling or adjustments in either age or isotope scales to maximize the higher resolution of the δ^{18}O data. The combined stratigraphy is verified by stratigraphically ordered ages by the independent chronologies. (upper panel).

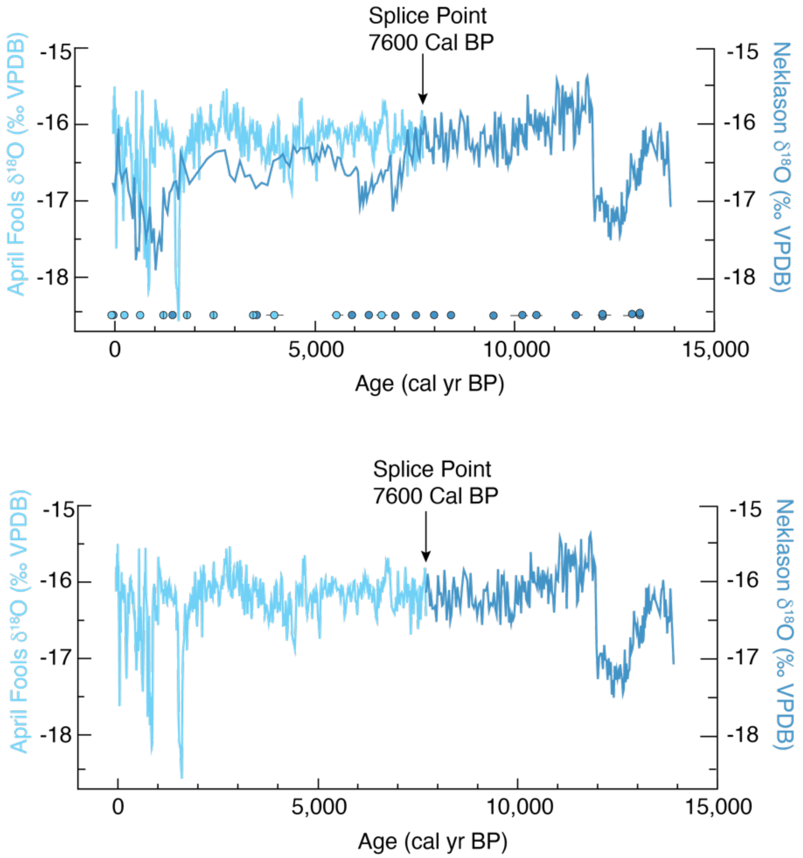

Supplementary Figure 7. Carbonate δ^{18}O records from April Fools core A15 (light blue, extending to 8ka) and Neklason Lake core B16 (dark blue) on a common per mil (‰) vertical axis with colored circles indicating age points for each core (upper panel). The records were combined at 7.6ka to form a single δ^{18}O stratigraphy (lower panel) with no vertical or horizontal scaling adjustments (lower panel).

Carbon and nitrogen isotopes of organic matter

Subsamples for organic carbon and nitrogen percentages and isotope analyses were acidified with 1 N HCL, rinsed to neutral, freeze dried and pulverized for combustion to CO_2 and NO_2 by a Carlo Erba™ CN elemental analyzer coupled with a Finnigan Delta™ Advantage isotope ratio mass spectrometer at the Idaho State University Stable Isotope Laboratory, Pocatello, ID, United States. Carbon isotope ratios are reported in per mil VPDB, defined by $\delta^{13}C = [(^{13}C/^{12}C)_{org} / (^{13}C/^{12}C)_{VPDB}] - 1$. Analytical precision for both carbon and nitrogen percentages, calculated from analysis of standards distributed throughout each run, is <0.5%. Analytical precision for $\delta^{13}C$ calculated from standards distributed throughout each run, is ±0.2‰.

The C/N ratio is often used to distinguish between terrestrial and aquatic sources[22], which can cause variability in organic $\delta^{13}C$ values. Terrestrial plants in Alaska, dominated by plants of the C3 photosynthetic pathway, tend to have higher C/N ratios relative to aquatic materials[23]. Factors that potentially control the $\delta^{13}C$ isotopic composition of aquatic materials include aquatic pCO_2 concentration, the $\delta^{13}C$ of DIC, and productivity[24]. Dissolved aquatic pCO_2 and DIC sources include atmospheric exchange and hydrologic input. Here we focus on interpreting factors controlling changes in aquatic $\delta^{13}C$ during a temporal window from the late deglacial to early Holocene period, when many Alaska lake records show a similar trend of decreasing $\delta^{13}C$ (Supplementary Fig. 8). This trend is unlikely to result from a change in organic source, as the C/N ratio is low (Supplementary Fig. 9), indicating aquatic material was relatively constant during this interval. Changes in aquatic plant $\delta^{13}C$ can result from input of DIC via watershed processes. A common observation in Alaska lakes is supersaturation of dissolved CO_2 in surface waters greatly depleted in ^{13}C relative to atmospheric sources resulting from decomposition of organic matter in lake watersheds[25] and subsequently transported to lakes via groundwater. This process leads to more negative aquatic plant $\delta^{13}C$ signatures. While changes in productivity can also influence aquatic $\delta^{13}C$, there is no systematic change in organic content across this interval (Supplementary Fig. 9). We conclude that increasing hydrologic input best explains declines in aquatic $\delta^{13}C$ in sedimentary records[26] following the end of the YD.

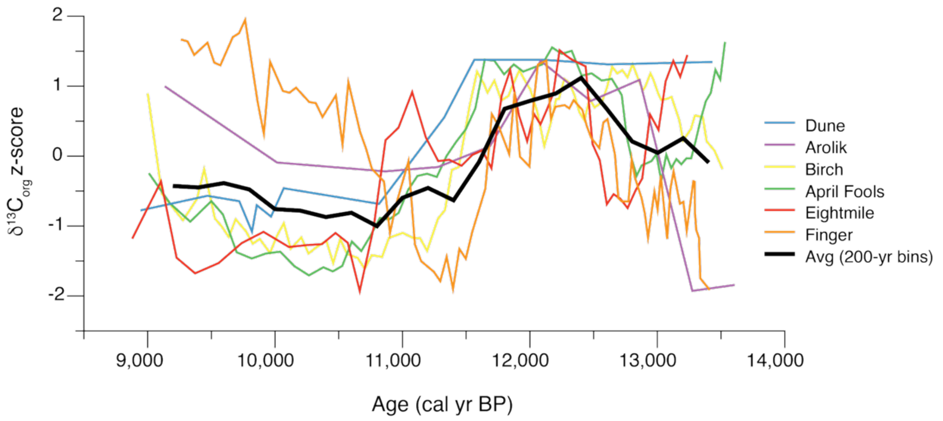

Supplementary Fig 8. Z-scores of Alaskan lake sediment $\delta^{13}C_{org}$ records between 9 and 14ka. Individual lake colored lines are indicated in the key. The thick black line is the average of 200-yr binned data from Birch, April Fools, Eightmile, and Finger (e.g., 9200 ka, 9400 ka...13,600ka). Data for Dune Lake and Arolik Lake are low in resolution and were not binned or included in the average calculation.

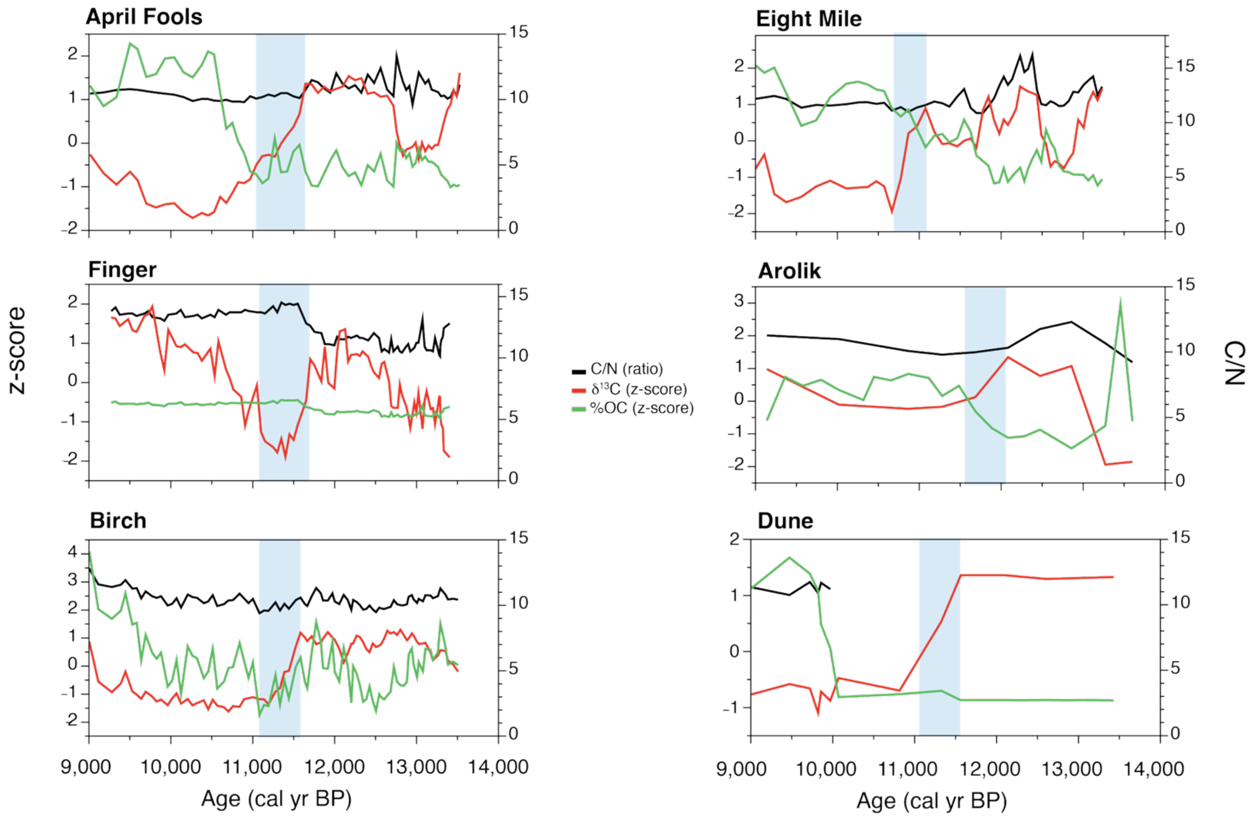

Supplementary Figure 9. Z-scores of Alaska lake organic sediment $\delta^{13}C$ and %OC are shown with C/N ratios between 9 and 14ka. Relatively low and invariant C/N (black, right vertical axis) indicate unchanging aquatic carbon sources during the periods of significant $\delta^{13}C$ decrease after ca. 11.7ka (red, left vertical axis). Organic carbon concentrations (green, left vertical axis) across the $\delta^{13}C$ decline are low and show 1) no change (Finger Lake, Dune Lake, April Fools Lake), 2) minor decline (Birch Lake), and 3) minor increase (Eight Mile Lake, Arolik Lake).

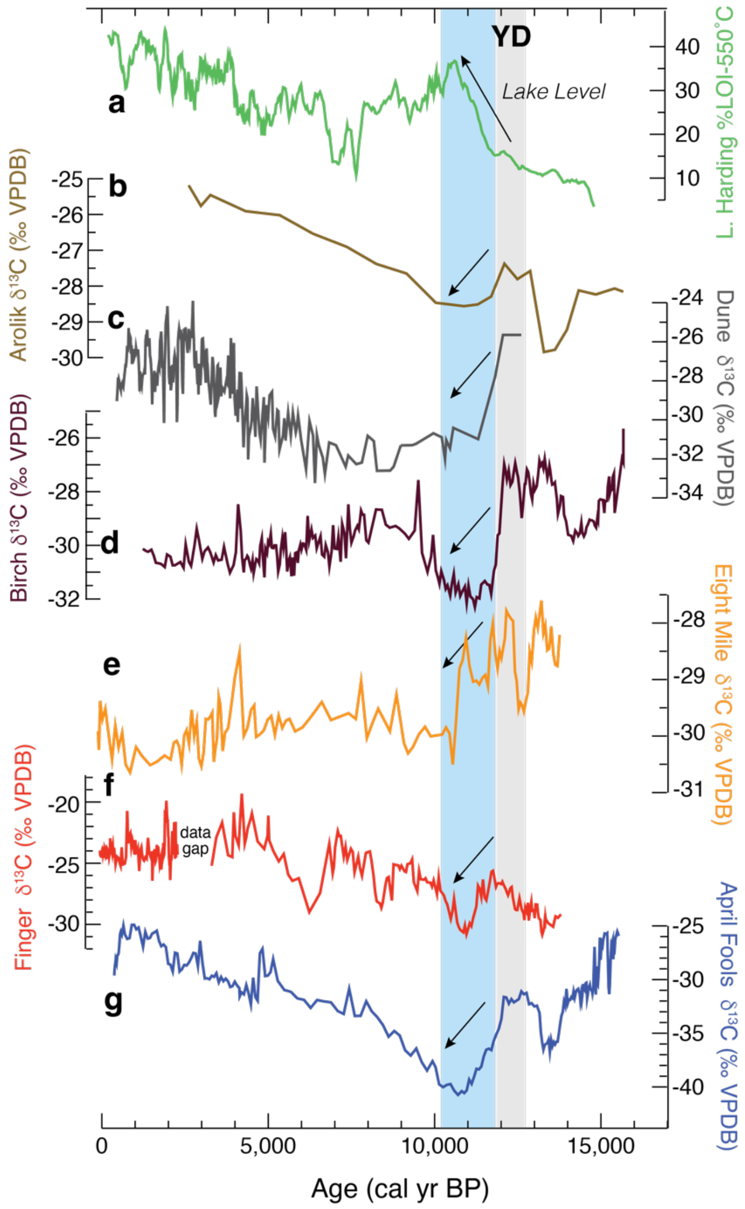

Supplementary Fig 10. Holocene records of Alaskan lake-level and organic sediment $\delta^{13}C$ **a,** organic matter concentrations for Little Harding Lake (green) correlated with a period of rising regional lake levels[19] (blue vertical shading) and **b - g,** organic sediment $\delta^{13}C$. **b,** Arolik Lake[15] (light brown), **c,** Dune Lake[26] (dark gray). **d,** Birch Lake (dark brown; this study). **e,** Eight Mile Lake[17] (light gray). **f,** Finger Lake (red; this study). **g,** April Fools Lake (dark blue; this study). Arrows highlight lake level rise and $\delta^{13}C$ declines that follow similar patterns in an interval following the YD (gray shading).

Supplementary Table 7: Radiocarbon data

[a] Not used in Bacon age-depth model
[b] v.8.2.1(IntCal20)

**Core Depth (cm)**	Material	^{14}C Date (^{14}C yr BP)	Measured Reservoir Offset	Reservoir Corrected ^{14}C Age (^{14}C yr BP)	AMS ^{14}C Lab #	δ^{13}C (‰ VPDB)	Median Calibrated Age (Cal yr BP)[b]	1σ range (Cal yr BP)[b]
**April Fools A15**								
0	Core top						-65	
28	^{210}Pb horizon						93	
59	Charred twig	655 ± 35			CAMS-176981		610	562-660
89	Aquatic herbaceous	4680 ± 25 [a]		1240	CAMS-177557	-19.2	1160 [a]	1123-1259
166	Aquatic herbaceous	5340 ± 60 [a]		1900	CAMS -177539	-21.8	1810 [a]	1736-1879
194	Aquatic herbaceous	5630 ± 30 [a]		2190	CAMS -177540	-17.8	2230 [a]	2145-2303
287	Aquatic herbaceous	6670 ± 30 [a]		3230	CAMS -177541	-20.1	3430 [a]	3399-3478
296	Tephra - Watana	3650 ± 150					3980	3765-4228
357	Conifer needle	4890 ± 70			CAMS -176979		5630	5491-5717
402	Tephra - Oshetna	5875 ± 85					6700	6563-6792
402	Aquatic herbaceous	9315 ± 35 [a]	3440	5875	CAMS -179998		6700 [a]	6663-6737
517	Leaf stem poss. aquatic	12380 ± 40 [a]		8940	CAMS -176980	-35.8	10060[a]	9959-10194
**April Fools B15**								
0	Core top						-65	
40	^{210}Pb horizon						93	
46	Wood	1110 ± 20[a]			NOSAMS-172587	-28.5	1010[a]	971-1054
245	Aquatic herbaceous	5425 ± 35		1985	CAMS-177542	-18.5	1915[a]	1878-1986
405	Tephra - Watana	3650 ± 150					3980	3724-4228
437	Wood	3925 ± 35			CAMS-177543	-29.7	4360	4295-4419
488	Conifer bract	5540 ± 20			CAMS-177544		6340	6295-6391
492	Tephra - Oshetna	5875 ± 85					6700	6563-6792
600	Juniper/Cassiopeia	8430 ± 140			CAMS-177545		9400	9150-9543
682	Aquatic herbaceous	13520 ± 70[a]		10080	CAMS-179999		11620[a]	11403-11812

709	Coal	35750 ± 790[a]	USGS-1503		>50,000[a]	NA
710	Wood	11940 ± 35	CAMS-176982	-27.9	13810	13762-13995
**Neklason A16**						
0	Core top				-66	
4	^{210}Pb horizon				93	
44	Wood	5530 ± 60	CAMS-180003		6330	6286-6394
52	Wood	6280 ± 35	CAMS-180034		7210	7165-7254
175	Wood	9375 ± 35	CAMS-180035		10600	10515-10657
286	Wood	12315 ± 35	CAMS-180004		14260	14153-14770
**Neklason B16**						
0	Core top				-66	
9	*Betula* seed and bract	Modern[a]	USGS-1891			
30	Broad leaf	1385 ± 25	USGS-1893		1300	1287-1308
48	Wood	3410 ± 30	NOSAMS-180036		3660	3614-3694
74	Wood	5150 ± 25	USGS-1894		5920	5901-5932
91	*Betula* sp. seed and scale	5600 ± 40	NOSAMS-172589		6370	6311-6404
101	*Betula* sp. seed & *Picea* sp. needle	6100 ± 25	USGS-1895	-27.5	6970	6905-7147
118	*Betula* sp. seed & *Picea* sp. bark	6745 ± 40	USGS-1896		7610	7574-7658
129	*Betula* sp. seeds & *Picea* sp. twig	7140 ± 30	NOSAMS-172590		7960	7935-8005
145	*Betula* sp. seeds & *Picea* sp. needles	7625 ± 30	USGS-1897	-25.1	8410	8385-8422
168	Wood	8395 ± 35	CAMS-180037		9440	9407-9477
196	Wood, broad leaf & bark	8965 ± 35	USGS-1898		10150	9964-10217
215	Wood	9430 ± 35	CAMS-180038		10660	10591-10706
260	Wood and broad leaf	10100 ± 70	NOSAMS-172591		11660	11498-11822
264	Bark (<0.2 mg)	8725 ± 50[a]	USGS-1899	-23.4	9690[a]	9555-9746
276	Wood	10425 ± 40	CAMS-180039		12300	12107-12582
280	Wood	10450 ± 75	NOSAMS-172592		12290	12264-12414
304	Wood	13120 ± 80[a]	NOSAMS-172593	-29.04	13120[a]	13064-13229

308	Wood	11025 ± 40	USGS-1901	-27.9	12960	12854-13059
313	Bark	11175 ± 35	CAMS-180042		13110	13086-13155
323	Charcoal	11160 ± 60	USGS-1902		13090	13003-13162
**Finger A16/23**						
0	Core top				-66	
22	^{210}Pb horizon				93	
48	Bark	430 ± 15	NOSAMS-172595	-28.0	500	494-506
112	Wood & broad leaf	1100 ± 15	NOSAMS-172596	-25.6	1000	958-1053
158	Broad leaf	1210 ± 20	NOSAMS-172597	-28.5	1125	1075-1164
226	Charcoal	1800 ± 170	CAMS-180053		1730	1535-1926
259	*Betula* sp. bract	2250 ± 40	NOSAMS-180229	-25.3	2230	2158-2336
270	*Betula* sp. seeds	2160 ± 25	NOSAMS-186571		2150	2109-2297
303	Wood	2640 ± 25	NOSAMS-189175		2755	2744-2761
345	Terrestrial unknown	3090 ± 45	NOSAMS-189176		3290	3241-3366
370	Charcoal	3400 ± 60	CAMS-180036		3650	3568-3719
377	Tephra – Watana	3650 ± 150			3980	3765-4228
415	Broad leaf & *Betula* sp. seed	4400 ± 40	NOSAMS-172599	-28.2	4970	4950-5040
438	Broad leaf, seed, wood	4710 ± 25	NOSAMS-180230	-27.7	5390	5328-5414
459	Broad leaf, seed, wood	4970 ± 35	NOSAMS-180231	-28.6	5690	5606-5730
482	Tephra – Oshetna	5875 ± 85			6700	6563-6792
492	Wood (<0.2mg)	6350 ± 30[a]	CAMS-180035		7280[a]	7257-7311
499	Wood	6245 ± 30	CAMS-180037		7200	7162-7245
512	Broadleaf	7060 ± 30	NOSAMS-180232	-28.0	7890	7855-7936
542	Wood and bark	8320 ± 35	NOSAMS-180233	-28.7	9350	9290-9419
556	Wood (<0.2mg)	8170 ± 75[a]	NOSAMS-172601	-27.4	9130[a]	9013-9143
595	Charcoal & broad leaf	10300 ± 65	NOSAMS-189177		12100	11889-12438
597	Charcoal	10250 ± 45	NOSAMS-180234	-26.5	11945	11829-12000
615	Alder/*Betula* sp. seed & insect	10650 ± 60	NOSAMS-189178		12670	12623-12724
627	Charcoal (<0.2mg)	10200 ± 55[a]	NOSAMS-180235	-27.1	11870[a]	11755-11945
640	Wood & broad leaf	11200 ± 220	NOSAMS-172602		13100	12895-13303
652	Insect	12600 ± 160	NOSAMS-180236	-27.8	14900	14498-15247

Supplementary References

1. McMillan, M. S. Spatial and temporal analyses of land cover, climate, and lake water quality in the Matanuska-Susitna Valley, Alaska. *M.S. Thesis Alaska Pacific Univ*. pp.66 (2015).

2. Gupta, P., et al. Demonstration of high-precision continuous measurements of water vapor isotopologues in laboratory and remote field deployments using wavelength-scanned cavity ring-down spectroscopy (WS-CRDS) technology. *Rap. Comms. Mass Spec.* **23**, 2534-2542 (2009).

3. Brand, W.A. et al. Cavity ring-down spectroscopy versus high-temperature conversion isotope ratio mass spectrometry; a case study on δ^2H and δ^{18}O of pure water samples and alcohol/water mixtures. *Rapid Commun. Mass Spectrom.* **23**, 1879-1884 (2009).

4. Bailey, H. L., Klein E. S., & Welker, J. M. Synoptic and mesoscale mechanisms drive winter precipitation δ^{18}O/δ^2H in South-Central Alaska. *J. Geophy. Res. Atmos.* **124**, 4252–66. (2019).

5. Kikuchi, C. P. Shallow groundwater in the Matanuska-Susitna Valley, Alaska - conceptualization and simulation of flow. *US Geo. Surv. Sci. Investig. Rep.* 2013-5049 (2013).

6. Dean, W.E. Determination of carbonate and organic matter in calcareous sediments and sedimentary rock by loss on ignition: comparison with other methods. *J. Sediment. Petrol.* **44**, 242-248 (1974).

7. Heiri, O. Loss on ignition as a method for estimating organic and carbonate content in sediments: reproducibility and comparability of results. *J.Paleolimnol*. **25**, 101-110 (2001).

8. Friedman, I. & O'Neil, J.R. Compilation of stable isotope fractionation factors of geochemical interest. In Fleischer, M (ed.) Data of Geochemistry, *US Geol. Surv. Prof. Pap.* 440-KK (1977).

9. Kim, S.T. & O'Neil, J.R. Equilibrium and nonequilibrium oxygen isotope effects in synthetic carbonates. *Geochim. Cosmochim. Act*. **61**, 3461-3475 (1997).

10. Leng, M.J. & Marshall, J.D. Palaeoclimate interpretation of stable isotope data from lake sediment archives. *Quat. Sci. Rev.* **23,** 811-831 (2004).

11. Appleby, P.G. Chronostratigraphic techniques in recent sediments. In *Tracking Environmental Change Using Lake Sediments: Basin Analysis, Coring, and Chronological Techniques*, Last, W.M. and Smol, J.P. (Eds) Kluwer Academic Press, Dordrecht, **1,** 171-203 (2001).

12. Bigelow, N.H. et al. Late-glacial paleoecology of the Middle Susitna Valley, Alaska: environmental context for human dispersal." *Front. Earth Sci.* **7**, 43 (2019).

13. Reimer, P.J. et al. The IntCal20 Northern Hemisphere Radiocarbon Age Calibration Curve (0-55 cal kBP). *Radiocarbon* **62**, 725-757 (2020).

14. Blaauw, M. & Christen, J.A. Flexible paleoclimate age-depth models using an autoregressive gamma process. *Bayesian Analysis.* **6**, 457-474 (2011).

15. Kaufman, D.S. et al. A ~33,000 year record of environmental change from Arolik Lake, Ahklun Mountains, Alaska, USA. *J. Paleolimnol.* **30**, 343-362 (2003).

16. Lynch, J.A. et al., Geographic and temporal variations in fire history in boreal ecosystems of Alaska. *J. Geophys. Res.* **108**, 8152 (2002).

17. Abbott, M.B. et al. Lake-Level reconstructions and paleohydrology of Birch Lake, Central Alaska, based on seismic reflection profiles and core transects. *Quat. Res.* **53**, 154–66 (2000).

18. Marshall, L. et al. 15,800 year record of productivity, carbon accumulation and environmental change at Eight Mile Lake and its catchment, central Alaska. *Arct. Antarct. Alp. Res.* **57**-2523083 (2025)

19. Brady, S.M. A multi-proxy approach to late Quaternary lake-level and environmental change at Little Harding Lake, interior Alaska. *Idaho State University M.Sc Thesis* (2013).

20. Faegri, K. Iversen, J. Textbook of Pollen Analysis. John Wiley and Sons, Chichester (1989).

21. Grimm, E.C. *Tilia* [Online]. Available at https://www.neotomadb.org/apps/tilia (2106).

22. Meyers, P.A. & R. Ishiwatari. Lacustrine organic geochemistry - an overview of indicators of organic matter sources and diagenesis in lake sediments. *Org. Geochem.* **20** 867–900 (1993).

23. Kling, G. W. et al. Arctic lakes and rivers as gas conduits to the atmosphere: implications for tundra carbon budgets. *Science* **251**, 298–301 (1991).

24. Laws E.A. et al. Dependence of phytoplankton carbon isotopic composition on growth rate and $[CO_2]_{aq}$: theoretical considerations and experimental results. *Geochim. Cosmochim. Act.* **59**, 1131–1138 (1995)

25. Campeau, A. et al. Stable carbon isotopes reveal soil-stream DIC linkages in contrasting headwater catchments. *J.Geophys. Res. Biogeosci,* **123** 149–167 (2018).

26. Finney, B.P. et al. Holocene climate change and carbon cycling in a groundwater-fed, boreal forest lake: Dune Lake, Alaska. *J. Paleolimnol*. **48**, 43-54 (2012).
